# Herbivory disrupts invasion-associated phyllosphere and arthropod community shifts in grasslands

**DOI:** 10.1016/j.isci.2026.117544

**Published:** 2026-09-15

**Authors:** Marco Fioratti Junod, Irene Cordero, Jennifer Firn, Sophie Gombeer, Gabrielle Lebbink, Martin Schütz, Anita C. Risch

**Affiliations:** 1 Biodiversity and Conservation Biology, Swiss Federal Institute for Forest, Snow and Landscape Research WSL, Birmensdorf, Switzerland; 2 Agroecology and Environment, Agroscope, Zurich, Switzerland; 3 School of Biology and Environmental Science, Queensland University of Technology, Brisbane, QLD, Australia

**Keywords:** grassland, invasion ecology, phyllosphere, trophic networks, land management

## Abstract

Biological invasions and changing herbivore communities can jointly reshape grassland ecosystems by altering vegetation structure, microclimate, microbial communities, and trophic interactions. We examined these effects in temperate Australian grassy woodlands dominated by kangaroo grass (*Themeda triandra*) and invaded by African lovegrass (*Eragrostis curvula*). Across six livestock-grazed farms, we established nested herbivore-exclusion treatments (open, livestock excluded, and all mammals excluded) in paired subsites dominated by kangaroo grass or co-dominated by African lovegrass. We measured microclimate, biomass, foliar damage, and characterized microbial phyllosphere and arthropod communities. The results revealed that African lovegrass invasion and herbivore exclusion independently and interactively reduced phyllosphere diversity, while concurrently increasing litter and lowering surface temperatures. Herbivore exclusion reorganized vegetation structure and invertebrate community composition, while shifting fungal infection profiles in ways that disproportionately affected the native grass. These results suggest that native mammals and livestock can partially buffer invasion-associated changes in ecosystem structure and biotic interactions.

## Highlights

• African lovegrass invasion reshaped phyllosphere microbes and arthropod assemblages
• Invasion and herbivore exclusion converged via litter accumulation and cooler soils
• Invaded plots showed reduced microbial diversity in kangaroo grass
• Moderate herbivory may limit litter build-up and invasion-associated community shifts

## Graphical abstract

**Figure:**
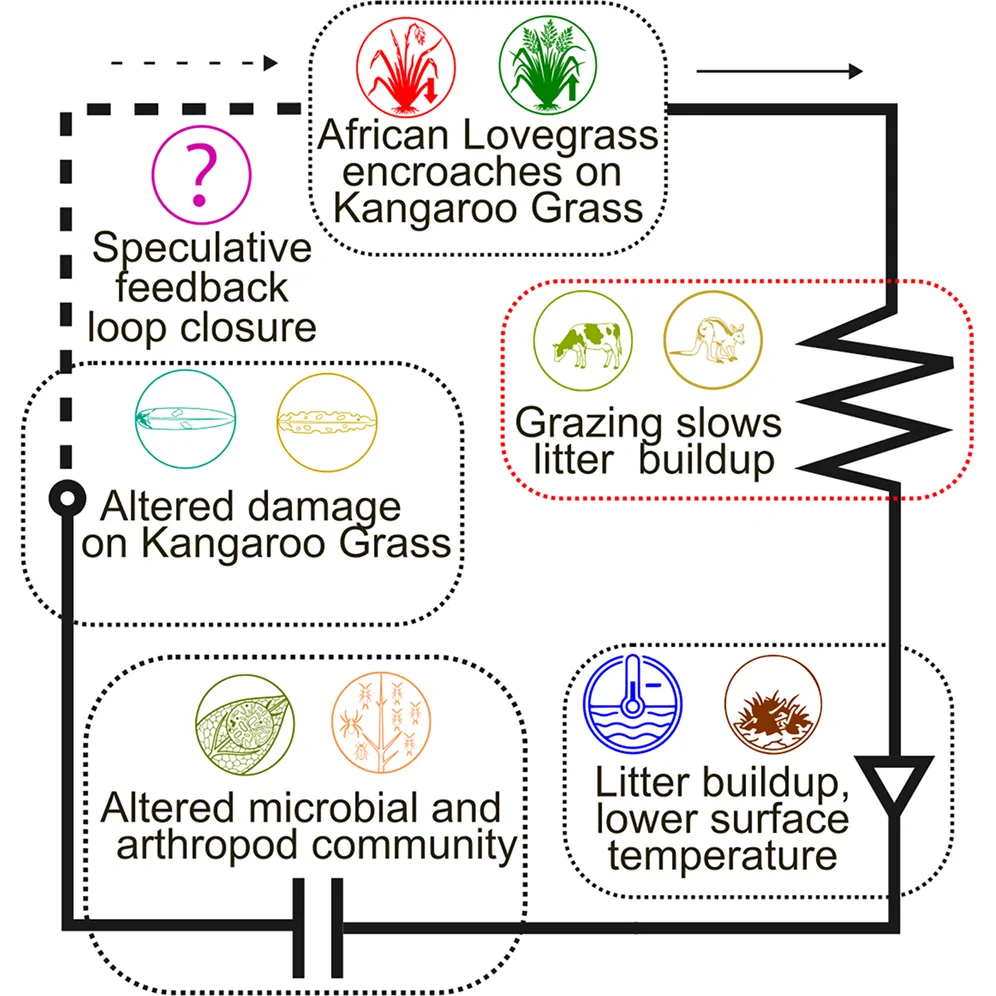

## Teaser

Ecology; Evolutionary ecology; Plant biology

## Introduction

Despite being rarely mentioned in sustainable development agendas,^1^ grassland ecosystems have a vital importance for the functioning of life on Earth. These ecosystems are essential for their role in carbon stock preservation,^2^ terrestrial food provisioning,^3^ non-agricultural mitigation and regulation services,^4^ and include some of the most biodiverse habitats on Earth.^5^ Invasive grasses are, however, increasingly impacting the provision of these ecosystem services across the globe.^6^^,^^7^^,^^8^ African lovegrass (*Eragrostis curvula* (Schrad.) Nees) and other closely related grass species are being perceived as a growing threat in all areas where they are not native.^9^^,^^10^^,^^11^^,^^12^

Pivotal to grassland health and functionality is the presence and abundance of microorganisms in the phyllosphere. In this study, we use the term phyllosphere to refer to the habitat formed by the surfaces of plant leaves. This habitat includes the physical space provided by the leaf surface and the fungal and bacterial communities that colonize it. We therefore use phyllosphere in a microbial and leaf surface-limited definition, excluding invertebrates as well as endophytic microbial communities living inside leaf tissues. The diversity of these leaf-surface inhabiting microbial communities is important for plant health and ecosystem functioning: their diversity has been linked to plant individual productivity and broader ecosystem function,^13^ as well as to resistance against pathogenic microbes.^14^

The composition of phyllosphere microbiota is shaped by a range of host-specific factors, including leaf surface microtopography and plant immune responses.^15^ While some studies suggest that the origin of a plant does not consistently shape its leaf communities,^16^ invasive plant species were found to often harbor distinct phyllosphere microbial communities that were intermediate between their native and introduced ranges.^17^ Invasive plant species can further impact phyllosphere and pathogen dynamics in native plant communities by serving as reservoirs for either novel^18^ or locally adapted pathogens. Where invasive plants are less susceptible to local pathogens, they can potentially gain a competitive advantage over native species—a mechanism described by the local pathogen accumulation hypothesis.^19^ The outcome of such pathogen-mediated interactions between native and invasive plant species may be taxonomically uneven.^20^

Invasive grasses can substantially alter plant community composition,^21^ soil properties,^22^ and fire risk,^23^ effects that may be associated with changes in aboveground communities and microbial assemblages over time. Although invasive plants may not disrupt food webs in grasslands as severely as in other ecosystems,^24^ this remains an understudied area.^25^

Vertebrate and invertebrate herbivores, which span multiple functional guilds and body sizes and act concurrently within a grassland ecosystem, are known to influence plant invasion dynamics,^26^^,^^27^ food webs based on detrital or living plant matter,^25^^,^^28^ the arthropod, and soil and foliar microbial community composition.^29^^,^^30^ The effects of different herbivore functional guilds on the phyllosphere are mediated by disturbance, competition, and changes in substrate heterogeneity. For instance, vertebrate grazers may reduce arthropod abundance while simultaneously increasing microbial diversity by affecting habitat complexity.^31^ Herbivory can also influence pathogen prevalence, either decreasing it through the consumption or physical removal of spores or increasing it by facilitating pathogen infection in the lesions generated on the plant leaves.^32^ The net effect of herbivory on the plant phyllosphere, therefore, is likely pathogen specific: biotrophic pathogens like rusts and powdery mildew may be suppressed, whereas necrotrophs such as leaf spot fungi could benefit.^33^ Nonetheless, evidence remains mixed and scarce. The few studies available show that the removal of invertebrate herbivores under experimental conditions has no effects on pathogen damage,^34^ while large ungulate exclusion has been observed to increase the diversity of fungal endophytes.^35^ These contrasting findings point to complex, potentially opposing effects of different herbivore functional and size guilds on phyllosphere microbial communities. The detrital food web also plays an important role, as surface-dwelling detritivores can react to different substrates by modulating the prevalence of pathogenic fungi.^36^

To deepen our understanding of the interaction between plant invasion, herbivory, and pathogen profiles, we established a field experiment on six farms in the Bega Valley, in southeastern New South Wales, Australia (see STAR Methods). The entire study area is a remnant of the threatened vegetation community known as lowland grassy woodlands.^37^^,^^38^ We established paired experimental exclosures at six sites, with one exclosure set up in an area dominated by native kangaroo grass (*Themeda triandra* Forssk) and one in an area co-dominated by non-native African lovegrass (*Eragrostis curvula*^39^) and kangaroo grass. The three-tiered experimental exclosures allowed access to different aboveground herbivore functional guilds, namely (1) livestock (cattle and sheep), rabbits (all non-native in Australia), native mammals, and invertebrates; (2) native mammals and invertebrates; and (3) invertebrates only. We assessed the combined effects of herbivory and plant invasion on (1) the phyllosphere microbial community composition and pathogen- and herbivory-induced damage on African lovegrass and kangaroo grass leaves, (2) surface- and vegetation-dwelling invertebrate communities and their functional guild structure, and (3) plant biomass accumulation and microclimate conditions at the soil surface. We tested the following hypotheses:

H1: African lovegrass leaves will host a different phyllosphere community compared to kangaroo grass,^15^^,^^40^^,^^41^ including a distinct pathogen profile.^42^

H2: Co-dominance with African lovegrass in invaded plots will be associated with changes in the phyllosphere and the pathogenic profiles of kangaroo grass leaves.^19^

H3: Herbivory will be associated with differences in the phyllosphere and pathogen-associated profiles of both target species, kangaroo grass and African lovegrass, with bigger expected differences for pathogens dependent on living tissue than for necrotrophic pathogens.^32^

H4: Herbivory, particularly by native large mammals and livestock, will be associated with changes in arthropod communities, including lower abundance of surface-dwelling arthropods and lower diversity of vegetation-dwelling insects. Based on previous work, we expected these patterns to be consistent with changes in resource availability and plant structural heterogeneity.^31^^,^^43^

H5: We do not expect substantial shifts in the relative representation of detritivore and herbivore arthropod functional guilds following African lovegrass invasion,^24^ but we predict that the number of surface-dwelling detritivores will be lower due to herbivory-induced litter reduction.^44^

## Results

### Plant damage scores

**Figure 1:**
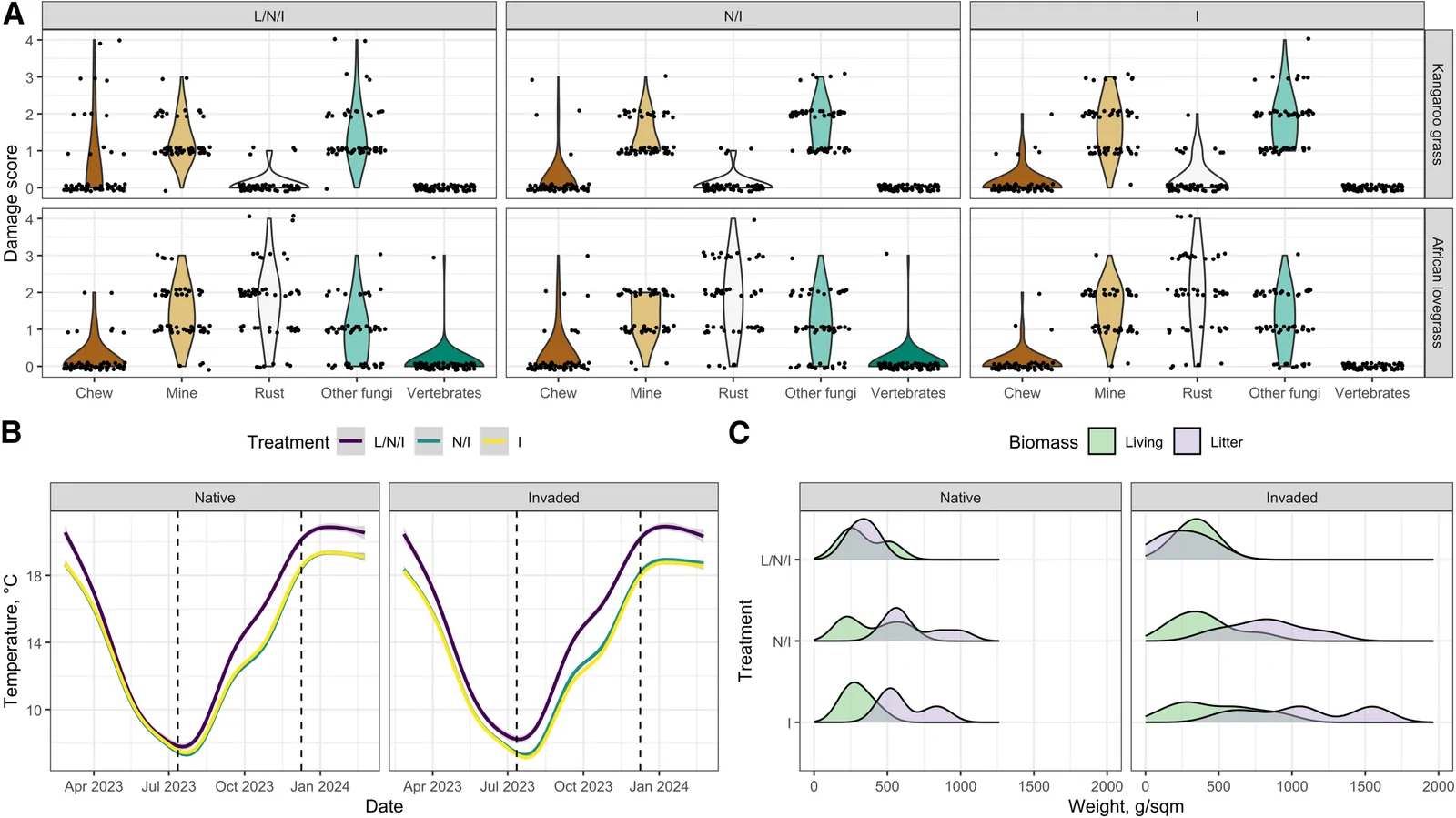
Plant damage, soil temperature, and litter (A) Plant damage score density plots for African lovegrass and kangaroo grass leaves assessed according to the guidelines defined by Castagneyrol et al.^45^ and Ebeling et al.^46^ Each point represents the score for an individual leaf, superimposed on the distribution density. (B) Smoothed conditional mean plot showing the evolution of temperatures 10 cm above the soil surface in the year preceding the sampling session. The two vertical dashed lines represent the seasonal breaking points used as knots used for splined modeling. (C) Density plot showing living and dead (litter) plant biomass (expressed as dry mass per m^2^) distribution recorded in the different exclosures, extrapolated from the combination of two sampled 20 cm by 100 cm strips.

Invertebrate chewing damage was significantly lower under mammalian herbivory exclusion in the N/I (livestock excluded) and I (all mammals excluded) treatments, resulting in a 46.1% (*p* = 0.022) and 73.7% (*p* < 0.001) lower plant damage score (Figure 1A) compared to the L/N/I (open) plots. Invasion did not result in significant changes in chewing damage, but African lovegrass leaves were overall less affected by chewing insects compared to kangaroo grass leaves, with a damage score lower by 40.3% (*p* = 0.037, Figure 1A). Mining damage did not vary significantly between the two grass species, invasion regime, or herbivory exclusion treatment. Instances of vertebrate damage were so few that the outcome could not be successfully modeled.

Fungal rusts did not differ significantly with herbivory exclusion treatment or plant invasion, but they affected African lovegrass overwhelmingly more than kangaroo grass and registered damage scores that were 18.2 times higher (*p* < 0.001) than on kangaroo grass (Figure 1A). The opposite trend was true for damage from other fungal pathogens (Figure 1A). Kangaroo grass was significantly more affected by these other fungal pathogens than African lovegrass, with a score that was 37% (*p* < 0.001) lower in African lovegrass. Damage by other fungal pathogens was also significantly higher with the exclusion of all mammals, i.e., the I treatment registered 29.2% (*p* = 0.025) higher scores compared to the L/N/I treatment. Both the N/I treatment and African lovegrass invasion showed near-significant (*p* = 0.098 and 0.097) higher values for these other fungal pathogens (Figure 1A) compared to the L/N/I and the native treatment.

### Plant biomass and surface temperature

The mean temperature recorded at 10 cm above the soil surface was 0.42°C (*p* = 0.048) lower in the N/I plot and 0.48°C (*p* = 0.025) lower in the I plot compared to the L/N/I treatment (Figure 1B).

The dry biomass of living plant tissue did not differ significantly among treatments, but the N/I and the I treatments had 2.85 (*p* < 0.001) and 2.53 (*p* < 0.001) times more dry litter per square meter compared to the L/N/I treatment (208 g). Invasion brought about an average 33% (*p* = 0.046) increase in dry plant litter (Figure 1C) compared to native plots.

### Phyllosphere microbial communities

**Figure 2:**
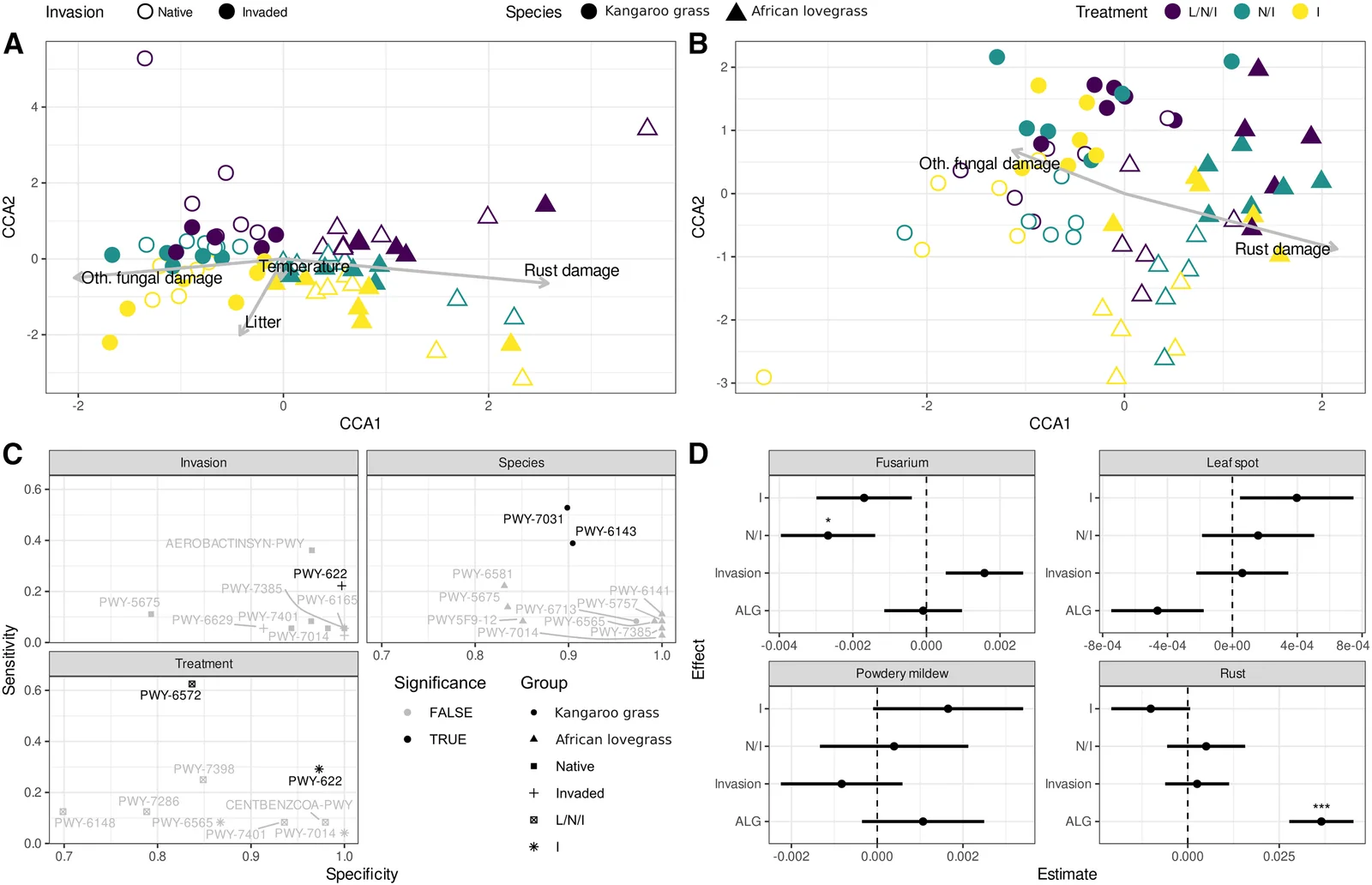
Microbial phyllosphere profiles (A and B) Canonical ordination of the (A) bacterial and (B) fungal phyllosphere communities according to grass species (African lovegrass and kangaroo grass), herbivory exclusion (L/N/I, N/I, and I), and invasion (native and invaded). Significantly correlated environmental vectors were overlaid. (C) Multilevel indicator analysis of the PICRUSt2-predicted pathways for the phyllosphere bacterial community. The axes are specificity and sensitivity. The image shows all pathways whose variation across patterns allowed computation of sensitivity and specificity scores. (D) Modeled effect sizes—with mean and confidence intervals—of predictors of the relative abundance of selected fungal clades associated with pathogenicity, namely *Fusarium*, *Pucciniales* (rusts), *Septoria* (leaf spot), and *Blumeria* (powdery mildew); ∗∗∗*p* < 0.001, ∗∗*p* < 0.01, ∗*p* < 0.05; *t* test.

Grass species (African lovegrass or kangaroo grass, *p* < 0.001) and invasion (*p* = 0.029) were significant factors in shaping the bacterial phyllosphere community. These differences in community composition were best explained by plant litter biomass, mean soil surface temperature, rust damage, and other fungal damage (*p* < 0.001, Figure 2A). Rarefied bacterial richness was lower in African lovegrass leaves compared to kangaroo grass (−21.7%, *p* < 0.001) and in invaded plots compared to the native ones (−9.5%, *p* < 0.001). Herbivory exclusion increased phyllosphere bacterial species richness significantly for both N/I (*p* < 0.001) and I (*p* = 0.043) treatments. However, the herbivory exclusion treatments had a negative compound interaction effect on bacterial richness on African lovegrass leaves (*p* = 0.020 and *p* < 0.001 for N/I and I, respectively). African lovegrass leaves also showed a significant reduction in bacterial Shannon’s diversity (−28.9%, *p* = 0.004) compared to kangaroo grass leaves.

Among bacterial families, *Cardiobacteriaceae* (*p* = 0.005), *Campylobacteraceae* (*p* = 0.005), *Listeriaceae* (*p* = 0.025), *Synergistaceae* (*p* = 0.035), *Nostocaceae* (*p* = 0.040), *Reynerellaceae* (*p* = 0.040), the groups WD2101 (order *Tepidisphaerales*, *p* = 0.040), and LWQ8 (order *Saccharimonadales*, *p* = 0.035) were associated with kangaroo grass leaves based on our indicator analysis. The family *Defluviicoccaceae* was associated with African lovegrass leaves (*p* = 0.045) the families *Bacteroidaceae* (*p* = 0.005), *Rikenellaceae* (*p* = 0.005), *Tannerellaceae* (*p* = 0.010), *Gemmatimonadaceae* (*p* = 0.005), *Erysipelotrichaceae* (*p* = 0.040), *Thermoactinomycetaceae* (*p* = 0.020), *Synergistaceae* (*p* = 0.015), and *Thermomonosporaceae* (*p* = 0.045) with native plots. Finally, the families *Synergistaceae* (*p* = 0.030), *Reyranellaceae* (*p* = 0.040), and *Micropepsaceae* (*p* = 0.035) were positively associated with the L/N/I treatment, and *Parachlamydiaceae* (*p* = 0.025) with the I treatment (see supplemental information, indicator bacteria).

The global PICRUSt2-derived functional profiles of our phyllosphere bacterial community, explored with a PERMANOVA (Permutational Multivariate Analysis of Variance) approach, did not structurally differ between the two grass species, invasion, or herbivory exclusion. However, indicator analysis on individual markers helped identify the following metabolic pathways from the MetaCyc taxonomy: pathways 6143 (*p* = 0.020) and 7031 (*p* = 0.005), involved in bacterial pathogenic adherence, were found to be indicators for kangaroo grass leaves; pathway 622, glycogen biosynthesis, was found to be an indicator for invaded plots and I plots (*p* = 0.005); pathway 6572, pertaining to degradation of carcasses and animal connective tissues, was an indicator for the L/N/I treatment (*p* = 0.030, Figure 2C).

Fungal phyllosphere communities significantly differed between the two grass species (*p* < 0.001), with invasion (*p* < 0.001) and herbivore exclusion (*p* = 0.040). Of all the supplementary environmental variables fitted onto the constrained ordination, only rust-induced damage and other fungal damage were significantly correlated (*p* < 0.001, Figure 2B) with the fungal phyllosphere communities. Fungal communities had significantly lower richness on African lovegrass leaves (−17.1, *p* < 0.001) compared to kangaroo grass leaves, invaded (−11.9%, *p* < −0.001) compared to native plots, but richness was higher in both N/I and I (*p* < 0.001) compared to the L/N/I plots. However, the effect of herbivory exclusion was reversed in African lovegrass leaves, with a significant negative effect (*p* < 0.001) on species richness. Shannon’s diversity of the fungal phyllosphere was also lower in African lovegrass leaves (−23.7%, *p* = 0.021) compared to kangaroo grass leaves and in invaded plots (−24.5%, *p* = 0.016) compared to native plots.

Among fungal orders, *Venturiales* (*p* = 0.005), *Entomophthorales*, *Saccharomycetales* (*p* = 0.005), *Orbiliales* (*p* = 0.025), *Lecanorales* (*p* = 0.015), and *Conioscyphales* (*p* = 0.025) were associated with kangaroo grass leaves; *Glomerellales* (*p* = 0.015), *Rhytismatales* (*p* = 0.030), and *Asterinales* (*p* = 0.035) with native plots; and *Microbotryales* (*p* = 0.005) with the L/N/I treatment (see supplemental information, indicator fungi).

As for fungal guild community composition, symbiotrophs, here referring to fungi that obtain resources through mutually beneficial associations with plant hosts, were significantly less abundant in I plots (−6.2%, *p* = 0.009), but no differences were detected between the leaves of kangaroo grass and African lovegrass. However, the African lovegrass phyllosphere also showed a marginally significantly (*p* = 0.053) higher proportion of plant pathogens. Rust-inducing fungi of the *Pucciniales* order were more abundant in the African lovegrass compared to the kangaroo grass phyllosphere (+3.65%, *p* < 0.001), whereas we found fewer *Fusarium* fungi in the herbivory exclusion treatments compared to the plots where all herbivores had access (L/N/I), although only in the N/I treatment was the effect significant (*p* = 0.041, Figure 2D). No significant grass species, invasion, and herbivory exclusion effects were detected for powdery mildew (*Blumeria*) or leaf spot (*Septoria*) pathogenic fungi (Figure 2D).

### Invertebrates

Invasion (*p* < 0.001), but not herbivore exclusion, was a driver of family-level community variation in surface-dwelling (pitfall collected) invertebrates. The opposite was observed for the vegetation-dwelling invertebrates (sweep net collected), where herbivore exclusion (*p* < 0.037) but not invasion was a significant driver of the invertebrate community composition.

**Figure 3:**
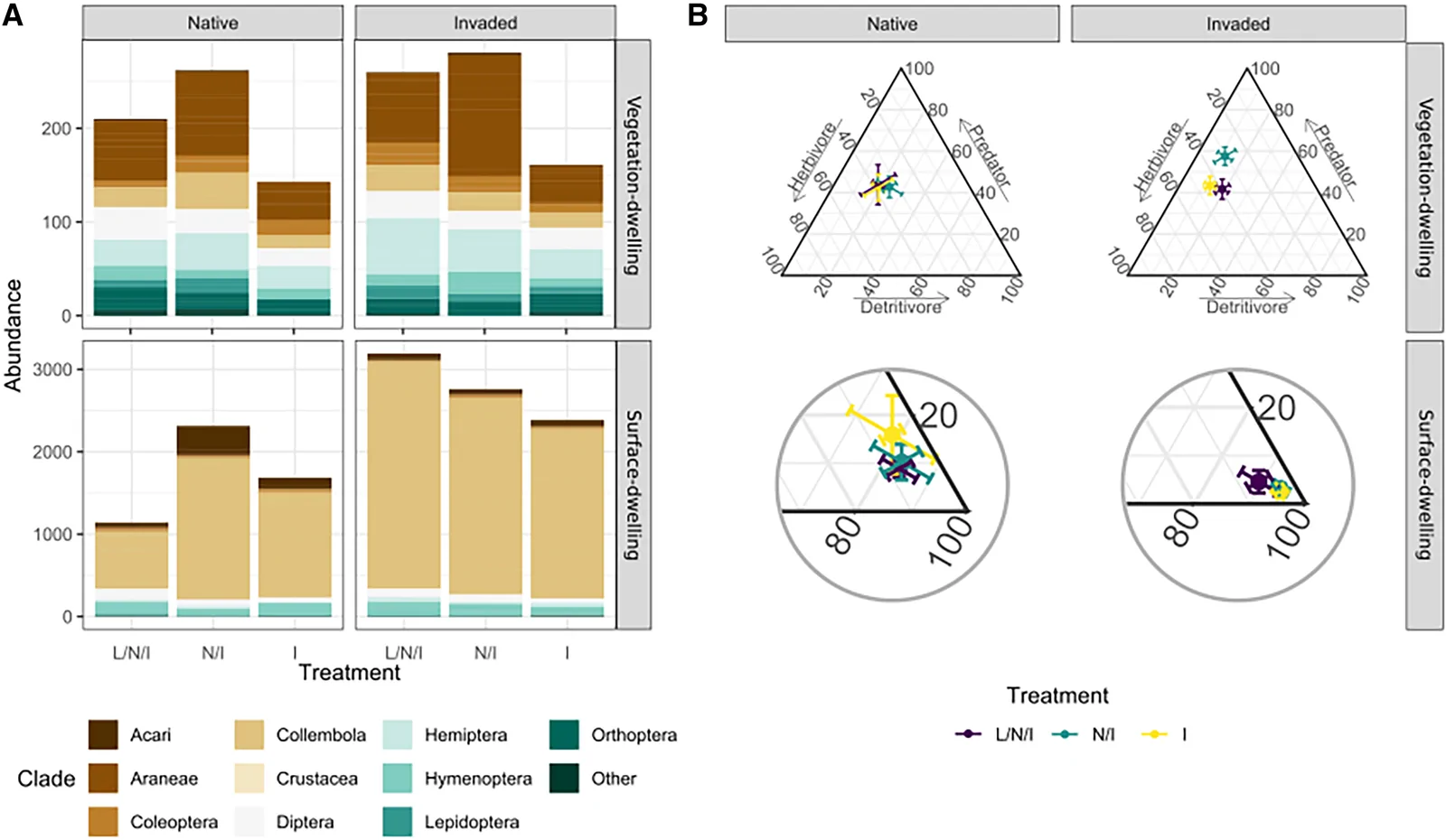
Invertebrate community profiles (A) Total abundance and taxonomic composition of invertebrate assemblages collected under each treatment combination, separated by sampling method: pitfall traps targeting surface-dwelling fauna and sweep-net transects targeting vegetation-dwelling fauna. Scale bars show cumulative abundance across replicates, partitioned by major taxonomic groups (see Clade legend). (B) Ternary diagrams illustrating the mean relative contribution (%) of three functional guilds (herbivores, detritivores, and predators) to invertebrate assemblages for each habitat type and sampling method. Points represent treatment means, with triaxial standard error bars indicating uncertainty around proportional estimates. Invertebrates were assigned to functional guilds at the family level based on published literature (see Data S1, guild table).

Surface-dwelling invertebrates showed significantly lower Shannon’s diversity in invaded plots compared to the native plots (−30.7%, *p* = 0.006). No significant differences in Shannon’s diversity were found for vegetation-dwelling invertebrates between native and invaded plots or between our herbivore exclusion treatments. Overall invertebrate abundance was significantly higher in invaded compared to native plots in both surface- (+62%, *p* < 0.001) and vegetation-dwelling invertebrates (+14.1%, *p* = 0.017, Figure 3A). As for herbivory exclusion, invertebrate abundance showed the same pattern for surface- and vegetation-dwelling invertebrates with higher numbers in the N/I treatment (+17.0%, *p* < 0.001 for surface-dwelling and +15.5%, *p* = 0.022 for vegetation-dwelling invertebrates, respectively) and markedly lower numbers in the I treatment (−6.2%, *p* = 0.004,-35.4%, *p* < 0.001) compared to the L/N/I treatment.

The guild-based community composition analysis (Figure 3B, see Data S1, guild table, for guild assignment) showed a negative impact of invasion on the relative abundance of predators (−70.3%, *p* = 0.012) and conversely a positive one for detritivores (+13.2%, *p* = 0.015) within the community of the surface-dwelling invertebrates. No significant effects of herbivory exclusion were found for the surface-dwelling invertebrate guilds, and no significant predictor emerged for the vegetation-dwelling invertebrate guilds.

### Herbivory/invasion/pathogen path analysis

**Figure 4:**
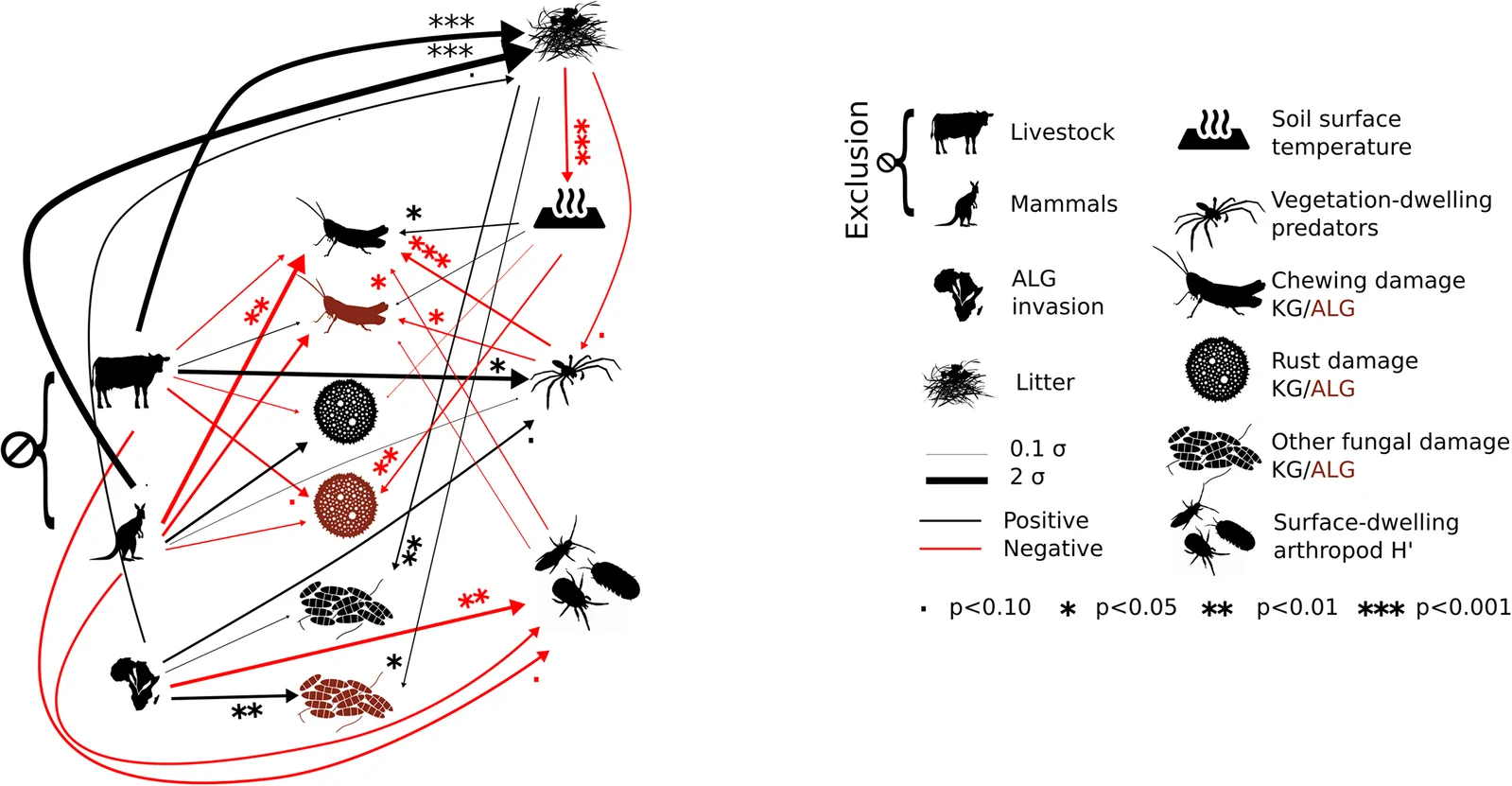
Path analysis derived from simultaneous regression models fitted in lavaan (R), using only observed variables and no latent constructs Model structure and directional paths were defined *a priori* based on published literature (Table S1). Arrows indicate hypothesized causal relationships underlying demonstrated correlations; arrow direction denotes the sign of the estimated standardized regression coefficient, and arrow width is proportional to its absolute magnitude. Symbols along paths indicate statistical significance levels (Wald’s z-test). The analysis accounts for the joint estimation of all paths, allowing evaluation of both direct and indirect effects within the specified causal network.

Both herbivore exclusion and African lovegrass invasion were associated with more plant litter, which in turn correlated negatively with solar irradiation and surface temperatures (Figure 4). The decrease in surface temperature was associated with an increase in the severity of rust damage on African lovegrass leaves, whereas plant litter accumulation was directly associated with an increase in other fungal damage on kangaroo grass leaves. Changes associated with both invasion and herbivory exclusion were correlated with lower diversity of surface-dwelling arthropods and with increased severity of chewing damage. Chewing damage was also strongly negatively correlated with invertebrate predator abundance, which in turn was positively correlated with livestock exclusion and African lovegrass invasion, but negatively with litter accumulation. The literature-informed model (supplemental information, Table S2) resulted in an RMSEA (Root Mean Square Error of Approximation) score of <0.001 and a comparative fit index >0.999, indicating an excellent fit.

## Discussion

We examined how African lovegrass invasion and the exclusion of mammalian herbivores independently and jointly relate to phyllosphere microbial communities, pathogen-associated damage and surface- and vegetation-dwelling arthropod assemblages. Our hypotheses (H1–H5) focused on the expectation that African lovegrass, as a recent invader of the investigated grasslands, would host distinct phyllosphere bacterial and fungal communities and pathogen-associated damage compared to the native kangaroo grass, and that proximity to African lovegrass would be associated with changes in the microbial community of kangaroo grass. We further predicted that herbivory would modulate both phyllosphere and arthropod community composition through its influence on plant biomass production and microclimate, with consequences for vegetation- and surface-dwelling arthropod functional guilds. Overall, our results showed that African lovegrass invasion and herbivore exclusion were associated with convergent changes, particularly via plant litter accumulation and associated microclimatic shifts. These alterations coincided with changes in pathogen-associated damage, microbial communities, and arthropod assemblages that could plausibly influence interactions and competition between African lovegrass and kangaroo grass. Later, we discuss these effects in detail, organized around the consequences of African lovegrass invasion, herbivore exclusion, and their ecological interactions.

### The consequences of African lovegrass invasion

As predicted in H1, the phyllosphere microbial communities differed considerably between kangaroo grass and African lovegrass leaves for both fungal (Figure 2B) and bacterial (Figure 2A) taxa. These differences were mirrored in the leaf damage scores, with distinct sets of fungal pathogens affecting the two grass species (Figures 1A and 2D). These results confirm previous findings that even closely related grass species can exhibit radically different pathogen damage profiles.^47^ In addition, the more severe chewing damage on kangaroo grass compared to African lovegrass may indicate the higher palatability of kangaroo grass for local invertebrate communities, perhaps due to their longer coevolutionary history.^48^ This pattern is consistent with the enemy release hypothesis, which proposes that invasive plants gain competitive advantage because they are less affected by local control factors, particularly phytophagous insects.^49^

African lovegrass invasion also led to an accumulation of plant litter on the soil surface (Figure 1C), which significantly lowered surface temperatures (Figure 1B) and likely increased surface humidity.^50^ These microclimatic changes contributed to an increase in the abundance of surface-dwelling detritivore invertebrates (Figure 3A, partly disproving H5) but a decrease in their overall diversity, a pattern consistent with meta-analytical evidence of a negative relation between abundance and diversity in decomposer assemblages.^51^ Moreover, we suggest that a subset of the original decomposer assemblage that co-evolved with native grasses will use invasive litter as a food source.

The effects of African lovegrass invasion extended to the phyllosphere of kangaroo grass (H2). The presence of African lovegrass was associated with a decrease in both fungal and bacterial diversity in African lovegrass and kangaroo grass leaves (Table S1). An invasive grass lacking a historical presence in the region restricts the pool of taxa able to colonize its phyllosphere to a limited number of generalists.^52^ This was especially evident in the lower prevalence of symbiotrophs on African lovegrass compared to kangaroo grass and in the reduced symbiotroph abundance in kangaroo grass leaves collected on invaded plots (H2, see Table S1). The depletion and homogenization of microbial communities are well documented in rhizosphere environments but remain little explored in the phyllosphere.^53^^,^^54^ Our findings suggest that similar processes of microbiome depletion and homogenization occur in the phyllosphere of invaded grasslands.

Such microbial reduction could contribute to the more severe damage from non-rust fungal diseases observed on kangaroo grass compared to African lovegrass (Figure 1A), because less diverse microbial communities may offer weaker protection against opportunistic pathogens.^55^ However, our study did not directly test pathogen transmission, microbial inhibition of pathogens, or demographic effects on either grass species. The observed reduction in foliar microbial and arthropod diversity associated with African lovegrass invasion, and particularly the decline of symbiotrophs in kangaroo grass when African lovegrass was present, therefore identifies a potential pathway through which invasion may affect native grass condition.^56^ Whether this pathway (see graphical abstract) feeds back to influence African lovegrass establishment, recruitment, or spread remains a hypothesis requiring further targeted tests.

### The consequences of herbivore exclusion

According to H3 and H4, herbivory exclusion was expected to influence both phyllosphere and arthropod communities. The most relevant microbial change linked to herbivore exclusion was the increase in bacterial richness accompanied by a decrease in fungal diversity. An increase in bacterial richness was previously reported in global grasslands with higher endophytic biomass,^35^ although fungal diversity also increased in those systems. In our case, this shift between fungal and bacterial taxa suggests a reorganization of the green biomass pathway, where a smaller number of primarily pathogenic fungal taxa dominate the foliar microbiome. Like invasion, herbivory exclusion led to an increase in plant litter and reduced surface temperature (Figures 1B and 1C), thereby favoring non-rust fungal infections in kangaroo grass (Figure 1A). These fungi likely benefited from the more humid microclimate generated by litter accumulation^57^^,^^58^ and from additional substrate to complete their necrophytic life cycles. In contrast, the prevalence of *Fusarium* spp. decreased under herbivory exclusion and was higher in invaded plots (Figure 1D). *Fusarium* includes many non-pathogenic strains; their presence can inhibit colonization by pathogenic forms.^59^ A shift in balance between pathogenic and non-pathogenic strains may, therefore, explain the increased damage by other fungal pathogens, affecting primarily kangaroo grass, observed under herbivore exclusion. Reduced herbivory was, therefore, associated with conditions under which non-rust fungal damage to kangaroo grass increased, but whether this translates into a competitive advantage for African lovegrass remains to be tested.

Unlike invasion, and opposite to what was predicted by H4 for surface-dwelling detritivore communities, herbivory exclusion did not alter the overall composition (Figure 3A). However, in agreement with what predicted by H4 for vegetation-dwelling arthropods, it was a significant predictor of their cmmunity composition. Previous studies had reported comparable trends between these two groups of invertebrates.^60^ In our experiment, chewing damage by arthropods decreased on both kangaroo grass and African lovegrass when all mammalian herbivores were excluded (Figure 1A). This may result from structural changes in the vegetation that enhanced predation pressure, particularly by spiders, on herbivorous insects such as orthopterans. Indeed, taller, denser vegetation generated by herbivory exclusion has been shown to increase predation rates of dummy caterpillars by arthropods^61^ and spider densities.^62^^,^^63^

### Interactions between grass invasion and herbivore exclusion and implications for conservation

Across all considered parameters in our study except bacterial taxon richness, the effect of grass invasion and herbivory exclusion affected the ecosystem in the same direction. The parallel effects of African lovegrass invasion and herbivory exclusion—both promoting plant litter accumulation—suggest a convergence of mechanisms that can amplify each other’s ecological impacts.

As anticipated, biotrophic pathogens such as rust fungi showed little change in prevalence in response to either African lovegrass invasion or herbivore exclusion, consistent with their reliance on living plant tissue.^34^ In contrast, necrotrophic fungal pathogens—especially those affecting kangaroo grass—were strongly associated with the combined effects of invasion and herbivory exclusion (Figure 1A). Plant litter accumulation, associated with African lovegrass presence and amplified under reduced herbivory (Figure 1C), coincided with more serious non-rust fungal damage on kangaroo grass. These results are consistent with a hypothesized link between invasion, herbivory, litter accumulation, and necrotrophic fungal damage,^33^ but they do not directly demonstrate pathogen spread or transmission.

The patterns we detected in the arthropod communities further reinforced this interaction framework. Increased arthropod numbers under livestock exclusion (Figure 3A) were consistent with resource competition between invertebrates and large mammals.^31^ However, when native mammals were also excluded, this trend reversed, possibly due to woody encroachment altering the physical environment.^64^ Vegetation-dwelling arthropods were more sensitive to herbivory exclusion, whereas African lovegrass invasion more strongly affected surface-dwelling communities, reducing their diversity through the accumulation of low-palatable litter.^65^

Overall, our findings show that plant litter accumulation following herbivore exclusion is associated with coordinated changes in arthropod and microbial communities that may intensify some ecological impacts of African lovegrass invasion. These patterns suggest a potential, but untested, pathway by which litter accumulation, fungal damage, and arthropod community shifts could skew the competitive balance between African lovegrass and kangaroo grass. Direct measurements of litter quality, decomposition, pathogen transmission, and long-term performance will be needed to determine whether these associations form a feedback that reinforces African lovegrass dominance over time.

Encouragingly, herbivory by both livestock and native mammals was associated with reduced litter accumulation and with less-pronounced shifts in pathogen-associated damage and arthropod community structure. By limiting standing biomass and litter build-up, moderate herbivory may weaken some invasion-associated community changes under low-herbivory conditions. Because livestock and native mammal herbivory produced largely consistent effects across measured variables, sustaining moderate herbivory in historically grazed grasslands^66^ may help maintain aboveground microbial and arthropod community structure.

### Limitations of the study

Because our study measured community composition, damage profiles, litter biomass, and microclimate, but not litter decomposition, pathogen transmission, energy fluxes, recruitment, or population balance between the two grass species, we interpret the proposed links as hypotheses about potential feedback pathways rather than as demonstrated demographic feedbacks. In addition, although phyllosphere responses were analyzed separately for kangaroo grass and African lovegrass wherever host identity was relevant, invasion necessarily entails a change in local plant composition; therefore, some plot-level differences associated with invaded subsites may partly reflect species turnover alongside invasion-associated changes in litter, microclimate and community structure.

## Resource availability

### Lead contact

Further information and requests for data and resources should be directed to and will be fulfilled by the lead contact, Marco Fioratti Junod (marco.fioratti@agroscope.admin.ch).

### Materials availability

This study did not generate new, unique reagents.

### Data and code availability

• The complete raw data supporting the present paper are available at Envidat: https://doi.org/10.16904/envidat.777.
• No original code was generated for this study.
• Any additional information required to reanalyze the data reported in this paper is available from the lead contact upon request.

## Acknowledgments

Funding was provided by an 10.13039/501100000923Australian Research Council Discovery grant DP190100500 “Managing complex networks in endangered grasslands to restore food webs”. Additionally, the authors would like to thank the farmers who allowed the establishment of the experimental setup on their properties and facilitated all the work involved and the two anonymous reviewers for their precious comments.

## Author contributions

J.F., A.C.R., and M.S. designed the experimental setup, obtained funding, and curated agreements and logistics. I.C., J.F., M.F.J., A.C.R., and M.S. conceptualized the project. M.F.J., S.G., G.L., M.S., and A.C.R. defined the fieldwork and sampling protocol and carried it out. M.F.J. and S.G. carried out the invertebrate sorting and identification and the molecular analyses. I.C. and M.F.J. analyzed and processed the data. M.F.J. wrote the first draft of the manuscript. I.C. and A.C.R. provided major revisions to the draft. I.C., J.F., M.F.J., S.G., G.L., M.S., and A.C.R. all reviewed and approved the manuscript.

## Declaration of interests

The authors declare no competing interests.

## STAR★Methods

### Key resources table

**Table:** 

REAGENT or RESOURCE	SOURCE	IDENTIFIER
**Deposited data**		

Data and code availability	EnviDat	Envidat: https://doi.org/10.16904/envidat.777

**Software and algorithms**		

R, version 4.4.2	R Core Team^67^	N/A
lme4 (1.1-35.3)	Bates et al.^68^	N/A
lmerTest (version 3.1.3)	Kuznetsova et al.^69^	N/A
emmeans( version 1.10.2)	Lenth^70^	N/A
hillR (version 0.5.2)	Li^71^	N/A
vegan (version 2.6.4)	Oksanen^72^	N/A
Indicspecies (vegan 1.8.0)	De Cáceres and Legendre^73^	N/A
PICRUSt2 77	Douglas et al.^74^	N/A
FUNGuildR (version.0.2.0.9, database 1.0)	Nguyen et al.^75^	N/A
lavaan	Roseel^76^	N/A

**Other**		

T-MS4 data loggers	Tomst, Prague, Czech Republic	N/A

### Method details

#### Site selection and experimental setup

In autumn 2019, we established herbivore exclosures on six active farms near Candelo, in the Bega Valley Shire of southeastern New South Wales, Australia (36°40' S, 149°40′ E, Figure S1). These farms are situated within lowland grassy woodlands characterised by a dominance of C4 grasses, other herbaceous plants, and red gum (*Eucalyptus tereticornis*,^37^). A primary ecological threat to these grasslands is African Lovegrass, an invasive grass species that is expanding globally.^38^^,^^77^^,^^78^ African Lovegrass poses a significant risk to native vegetation due to its allelopathic effects,^79^ rapid early growth,^80^^,^^81^ and ability to thrive in nitrogen-enriched soils.^82^ One of its key native competitors is Kangaroo Grass, a C4 perennial tussock grass with medium to low palatability for livestock and a growth form adapted to fire regimes.^12^

Each farm contained two subsites located within 100 m from one another on comparable soil types, expositions and slopes: one subsite was classified as “native,” where Kangaroo Grass was dominant, the other classified as “invaded,” where Kangaroo Grass coexisted with African Lovegrass. Both grass species were present in all plots. The farms were managed under extensive grazing by livestock (chiefly cattle and sheep). African Lovegrass control strategies varied between farms and included roller wiping, slashing, or no intervention. Both native (e.g., kangaroos, wallabies, wombats) and non-native wild mammalian species (e.g., rabbits) as well as livestock and invertebrates had unrestricted access to these farms.

We established a nested exclosure system^57^ within each subsite in the early summer of 2019 and 2020 (with delays experienced due to extreme fire events in the region) to regulate access by the different herbivore groups (Figure S2):

L/N/I (20 × 20 m): An open, unfenced area allowing access to livestock (L), native mammals (N), and invertebrates (I), positioned at least 5 meters from the adjacent exclosure.

N/I (20 × 20 m): Excluded livestock and rabbits while permitting access to native mammals (N) and invertebrates (I). This exclosure featured wooden posts and non-barbed wire positioned at 20, 40, 80, and 130 cm heights. A 20-mm mesh extended up to 40 cm above the ground and was angled 20° below the surface to prevent rabbit burrowing. Wallaby and kangaroo scat was recovered in the N/I exclosure at comparable densities to L/N/I, showing access by large Macropodidae was not impeded and that these animals could access the exclosure by jumping over the fence.

I (5 × 5 m): Nested within the N/I exclosure, this plot allowed access for invertebrates only (I). The fencing followed the same design as the N/I exclosure but extended the mesh to a height of 150 cm, thereby preventing entry by wombats and wallabies due to physical barriers and excluding kangaroos by limiting available space.

All data were collected on 19–21 and 26–28 February 2024.

#### Individual plant species assessment

In each exclosure, five mature leaves from the two target grass species, Kangaroo Grass and African Lovegrass, were randomly selected. The blade was examined for damage evidence of the following categories: insect chewing and mining, rust, mildew (not detected), other fungal damage and vertebrate biting. Damage severity (0 = no damage; 1 ≤ 1%, 2 = 2-25%; 3 = 26-50%, 4 = >50% of the blade surface; hereafter plant damage scores) was recorded for each damage type according to the protocol detailed by Castagneyrol et al.^45^ and Ebeling et al.^46^

Another three mature leaves of the two grass species were randomly selected in every exclosure plot and a sterile swab (Swab Collection and DNA Preservation System, Norgen Biotek, Norold, Canada) was rubbed for 30 seconds against the upper and lower leaf surface of each blade. The swab was then sealed in the preservative-filled vial part of the same kit and stored at 4°C until further processing. Two field blank swabs were opened and transferred to the preservative vial on site, without contact with blade surfaces. DNA was extracted with a commercial kit (DNeasy® Powersoil®, Qiagen, Hilden, Germany). The terminal (coated) part of the swab together with 250 μL of the preservation solution were loaded in the lysis column of the kit. Afterwards, we followed the DNA isolation and purification protocol according to the manufacturer's instructions. Two technical blanks were obtained by processing the lysis columns without sample addition. The DNA yield was measured using the Qubit™ dsDNA High Sensitivity kit and instrument (ThermoFisher, Waltham, USA). The extracted DNA samples were sent to Novogene Ltd (UK) for amplification and sequencing using an Illumina paired-end 250 bp platform. A fragment of the hypervariable V4 region of the 16s rRNA gene was amplified with the primer pair 515F/806R (GTGCCAGCMGCCGCGGTAA/GGACTACHVGGGTWTCTAAT, adapted from literature^83^ for prokaryotes. For fungi the primer pair ITS3-2024F/ITS4-2409R (GCATCGATGAAGAACGCAGC/TCCTCCGCTTATTGATATGC, adapted from literature,^84^ amplifying a portion of the ITS2 region, was used. Denoising, chimera removal and clustering were carried out with the QIIME pipeline allowing to retain 97% (16S) and 90% (ITS2) of reads. OTUs present with more than five reads in any of the blanks were discarded, resulting in a total of 4444 bacterial and 3614 fungal OTUs. Taxonomic assignment was performed against the SILVA (version 138.2) and Unite (version 10.0) databases for bacteria and fungi respectively. Inferred EC (Enzyme Commission,^85^) functional profiles and pathways for bacteria were extracted with the PICRUSt2 pipeline.^74^ For fungi, guild and trophic mode information was extracted with the FUNGuildR package in R (version 0.2.0.9, first published database^75^).

#### Plant live and dead biomass measurements

Two randomly selected strips (20 × 100 cm each) were identified within each experimental plot across all subsites. All aboveground vegetation was clipped with secateurs as close to the ground as possible and collected in paper bags, separating live biomass from plant litter, here defined as dead biomass, independent of it being still attached to a living plant. The samples were then dried at 60°C for 48 hours and immediately weighed to determine dry biomass (g). The values from the two strips were averaged and scaled to a per-square-metre basis.

#### Pitfall-trapping and sweep-netting

In each exclosure, a pitfall trap was deployed and left in operation for one week before collection. The pitfall traps consisted of plastic tubes (13 cm depth and 6.75 cm diameter) that were inserted into the ground after a hole of the same size was punched with a soil corer. The upper rims of the tubes were flush with the soil surface and contained funnels with the same diameter directing invertebrates to the underlying 100 ml collection vials containing 75 ml of 20% propylene glycol. As no substantial precipitation was expected during the operating time, no cover was installed. The preservative was changed to 75% methylated spirits after sample collection for downstream sorting and identification (see below).

In each exclosure, two sweep-netting sessions were carried out, one week apart. The sessions covered an area of ∼25 square metres, with a lateral sweep width of ∼1 m and a non-linear transect of 25 m. In the case of the I exclosure, the session therefore covered the entire area of the exclosure. All transects were walked at constant pace by the same surveyor. The heavy duty, 400 μm, professional sweep-net (NHBS, Totnes, UK) was swept as close as feasible to the soil surface during collection. The catches were immediately transferred to a 100 ml vial filled with 75% methylated spirits. The two catches from the same exclosure were combined prior to sorting.

The content of vials obtained with pitfall trapping (hereafter surface-dwelling invertebrates) and sweep netting (hereafter vegetation-dwelling invertebrates) was transferred to Petri dishes, sorted and identified under a dissection microscope whenever possible to family level.^86^^,^^87^^,^^88^^,^^89^^,^^90^ Functional guilds were assigned to family level based on literature (see Data S1, Guild table).

#### Soil surface temperature measurements

A T-MS4 data logger (Tomst, Prague, Czech Republic) was installed in each treatment plot within every subsite at the start of the experiment and remained in place throughout its duration. The logger recorded temperature at 10 cm above the soil surface at 15 minute-intervals during the timeframe considered.

### Quantification and statistical analysis

The effects of herbivore exclusion treatments on continuous variables were tested by fitting mixed effects linear models (LMMs; lme4 package version 1.1-35.3^68^). Herbivory exclusion (categorical variable with 3 factors) and invasion (binary) were fixed explanatory variables and farm a random factor. For count variables (e.g., number of species, number of specimens or damage scores), an underlying Poisson distribution was assumed. In addition, for modelling plant damage scores, a random factor for exclosure to account for the multiple leaves collected for the measurements, in addition to farm, was included. Extraction of treatment effects and marginal means was performed with the packages lmerTest (version 3.1.3^69^) and emmeans (version1.10.2^70^). Shannon’s diversity indices and species richness for the invertebrate communities were calculated with Hill’s numbers with the R package hillR (version 0.5.2^71^). A summary table of the models fit for each variable is presented as Table S1.

Permutational analysis of variance, canonical correspondence analyses and environmental vector fitting on community data (bacteria, fungi, invertebrates) were performed with the functions adonis2, cca and envfit of the R package vegan (version 2.6.4,^72^^,^^91^). For canonical correspondence analyses of the phyllosphere, the model included the species (African Lovegrass or Kangaroo Grass), herbivory exclusion, invasion and farm. For invertebrates, as data are not species specific, the model only included herbivory exclusion and invasion in addition to farm.

Multilevel indicator analyses to identify candidate indicator taxa for invertebrates, fungi, bacteria were performed with the multipatt function of the R package indicspecies (1.8.0^73^) separately for plant species, herbivory exclusion level and invasion. Multilevel analyses with the same method were applied to PICRUSt2 derived pathways, with the separate components of specificity and sensitivity extracted for visualisation.

Path analysis applied to plant/animal/pathogen interactions was performed with the function fit from the R package lavaan (version 0.6.17,^76^). A selection of variables measured across all samples and their interactions was made according to existing literature (supplemental information, Table S2) to test the direction and strength of functional guild herbivory and invasion on pathogen profiles directly or through the filter of microclimate variables, predatory invertebrates controlling chewing damage and detritivore invertebrates within the litter layer. The variables were fit into a regression network, after standardisation.

A two-knot splined linear model was fitted to the data for surface temperature in the year preceding the sampling, measured at 10 cm above the surface, to take into account seasonal breaking points on the 12th of July and 9th of December 2023, invasion and herbivory exclusion as explanatory variables and farm as random effect. For every day, a single input value was obtained by averaging all temperature values collected at 15-minute intervals throughout the day.

Significance levels provided are the result of two-sided t-tests except for environmental variable fitting and permutational multivariate analysis of variance, where it is expressed in permutations, and path analysis, where it is the result of Wald’s z-tests.

All analyses, as well as data cleaning and processing, were performed with R version 4.3.2^67^ in an RStudio Chocolate Cosmos release^92^ environment.

## Supplemental information

Document S1. Figures S1 and S2 and Tables S1 and S2

Data S1. Guild table includes the assignment of invertebrate taxa to feeding guilds based on established literature
