# Supplementary material for: Herbivory disrupts invasion-associated phyllosphere and arthropod community shifts in grasslands
Source: iScience. 2026 Sep 15;29(10):117544. doi: 10.1016/j.isci.2026.117544 (PMC13595126; doi:10.1016/j.isci.2026.117544)
Supplement: Supplementary file 1 [file mmc1.pdf]

iScience, Volume *29*

Supplemental information

Herbivory disrupts invasion-associated phyllosphere and arthropod community shifts in grasslands

Marco Fioratti Junod, Irene Cordero, Jennifer Firn, Sophie Gombeer, Gabrielle Lebbink, Martin Schütz, and Anita C. Risch

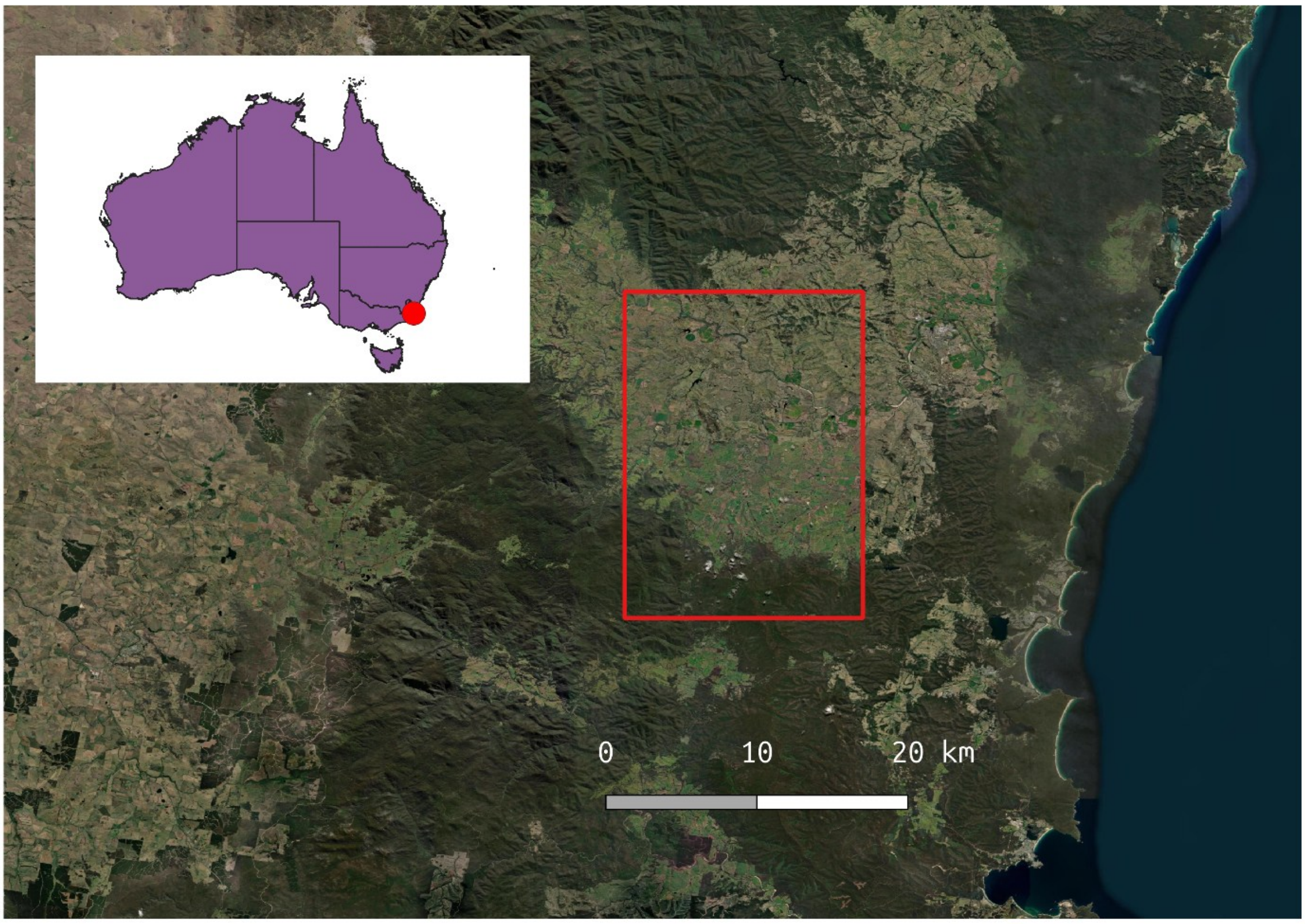

Figure S1: Satellite image of the study area in the Bega Valley Shire and positioning within Australia.

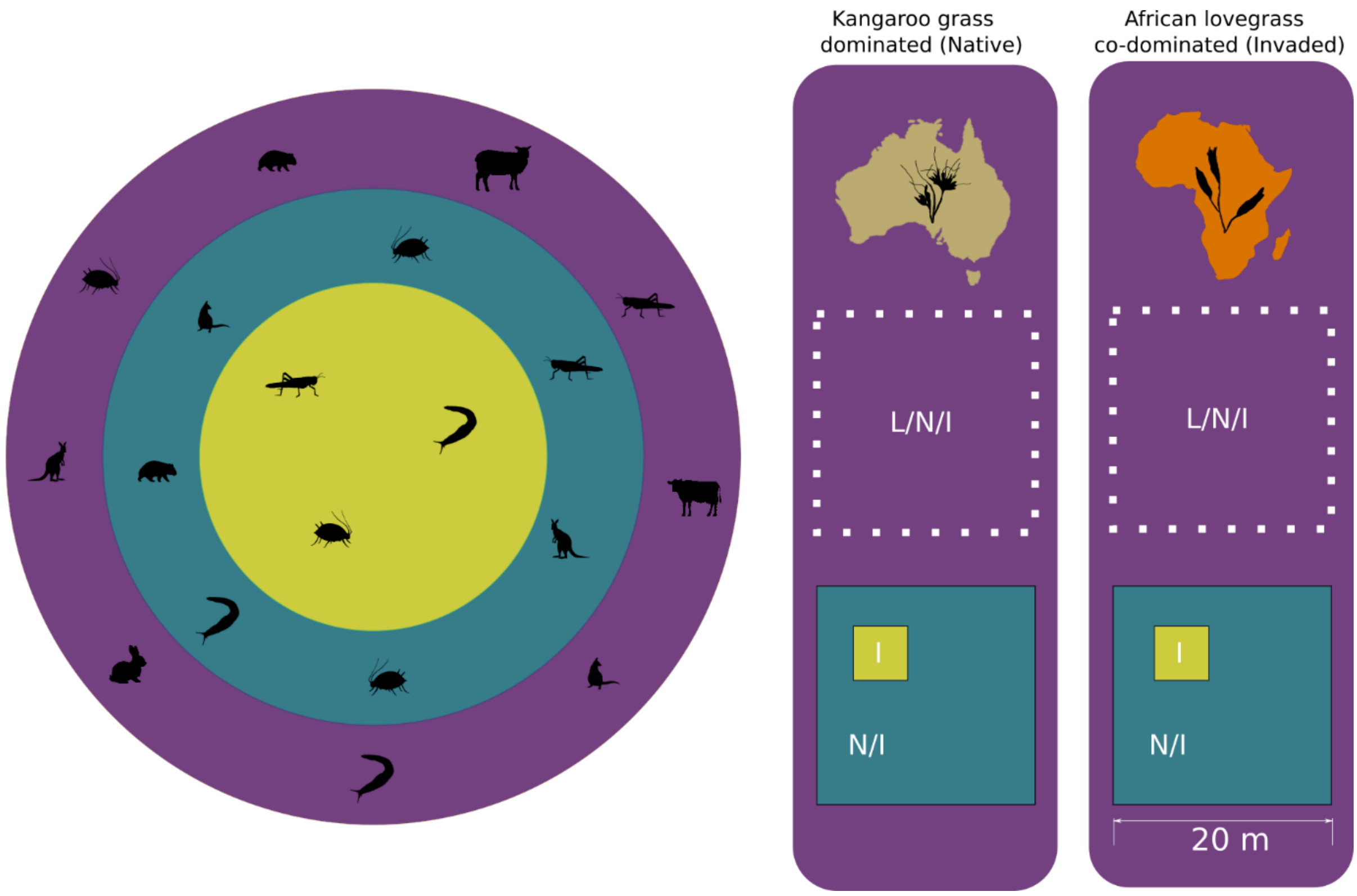

Figure S2: Conceptual diagram of the experimental exclosure layout, adapted from Fioratti Junod et al. (2024)

Table S1: Summary statistics table. The data shows the effect size estimates and significance .for each of the predictor variables fitted to each model. Where interaction effects between species and treatment were significant, they were retained in the model and the output shown hereafter.Intercept values refer to the baseline defined as the L/N/I treatment in native plots (and in Kangaroo Grass for leaf-specific variables).

Response	Intercept	N/I	I	Invasion	ALG	ALG×N/I	ALG×I	Distribution
Chewing damage score	-1.26	-0.62 ± 0.27 (.022)	-1.34 ± 0.35 (<.001)	0.107 ± 0.24 (.656)	-0.516 ± 0.25 (.037)	—	—	N
Rust damage score	-2.46	0.04 ± 0.14 (.786)	0.16 ± 0.13 (.220)	0.174 ± 0.11 (.113)	2.897 ± 0.24 (<.001)	—	—	N
Other fungal pathogens damage score	0.26	0.19 ± 0.12 (.097)	0.26 ± 0.11 (.025)	0.150 ± 0.09 (.098)	-0.458 ± 0.09 (<.001)	—	—	N
Mining damage score	0.23	0.01 ± 0.11 (.913)	0.15 ± 0.11 (.913)	0.151 ± 0.11 (.150)	0.025 ± 0.09 (0.780)	—	—	N
Soil surface temperature	16.03	-0.42 ± 0.20 (.048)	-0.48 ± 0.20 (.025)	-0.039 ± 0.16 (.810)	—	—	—	N
Living biomass, grams per square metre	307.78	60.29 ± 74.40 (.425)	52.98 ± 74.40 (.482)	61.730 ± 60.75 (.319)	—	—	—	N
Litter biomass, grams per square meter	207.74	455.42 ± 106.36 (<.001)	553.61 ± 106.36 (<.001)	179.940 ± 86.84 (.046)	—	—	—	N
16s richness (rarefied)	5.91	0.11 ± 0.21 (<.001)	0.04 ± 0.02 (.043)	-0.102 ± 0.01 (<.001)	-0.170 ± 0.02 (<.001)	-0.073 ± 0.031 (.020)	-0.121 ± 0.032 (<.001)	Poisson
16s diversity (rarefied)	62.327	2.857 ± 7.486 (.704)	2.771 ± 7.486 (.713)	-0.474 ± 6.11 (.938)	-18.456 ± 6.11 (.004)	—	—	N
ITS richness (rarefied)	6.04	0.17 ± 0.02 (<.001)	0.17 ± 0.02 (<0001)	-0.13 ± 0.01 (<.001)	-0.09 ± 0.02 (<.001)	-0.11 ± 0.03 (<.001)	-0.17 ± 0.03 (<.001)	Poisson
ITS diversity (rarefied)	60.222	-3.321 ± 6.521 (.613)	4.679 ± 6.598 (.481)	-13.254 ± 5.35 (.016)	-12.809 ± 5.38 (.021)	—	—	N
Symbiotrophs (relative abundance)	0.227	-0.034 ± 0.023 (.147)	-0.063 ± 0.023 (.009)	0.015 ± 0.02 (.009)	-0.023 ± 0.02 (.235)	—	—	N
Plant pathogens (relative abundance)	0.345	0.028 ± 0.029 (.328)	-0.032 ± 0.029 (.328)	-0.006 ± 0.02 (.791)	0.047 ± 0.02 (.053)	—	—	N
Soil dwelling invertebrate richness (per pitfall)	2.91	-0.15 ± 0.10 (.144)	-0.03 ± 0.10 (.769)	-0.09 ± 0.08 (.288)	—	—	—	Poisson
Soil dwelling invertebrate abundance (absolute, per pitfall)	5.42	0.16 ± 0.02 (<.001)	0.06 ± 0.02 (.004)	0.48 ± 0.02 (<.001)	—	—	—	Poisson
Soil dwelling invertebrate diversity (per pitfall)	1.998	-0.363 ± 0.224 (.117)	-0.257 ± 0.224 (.260)	-0.547 ± 0.18 (.006)	—	—	—	N
Above-ground invertebrate richness	2.71	0.02 ± 0.10 (.877)	-0.21 ± 0.11 (.051)	0.04 ± 0.09 (.632)	—	—	—	Poisson
Above-ground invertebrate abundance (absolute)	3.54	0.14 ± 0.06 (.022)	-0.44 ± 0.07 (<.001)	0.13 ± 0.06 (.017)	—	—	—	Poisson
Above-ground invertebrate diversity	2.348	-0.106 ± 0.111 (.348)	-0.099 ± 0.111 (.383)	0.030 ± 0.09 (.743)	—	—	—	N

Table S2: Theoretical background and literature on which we built the a-priori paths identified for the structural equation model.

Upstream	Downstream	Reference
Herbivory exclusion	*Litter*	(Su et al. 2022)
Invasion	*Litter*	(Csurhes et al. 2016)
Litter	*Temperature*	(Hou et al. 2019)
Litter	*Predators*	(Uetz 1979)
Herbivory exclusion	*Predators*	(Vandegehuchte et al. 2017)
Invasion	*Predators*	(Litt et al. 2014)
Herbivory exclusion	*Invertebrate diversity*	(van Klink et al. 2015)
Invasion	*Invertebrate diversity*	(Litt et al. 2014)
Herbivory exclusion	*Chewing damage*	(van Klink et al. 2015)
Temperature	*Chewing damage*	(Fricke et al. 2022)
Predators	*Chewing damage*	(Unsicker et al. 2006)
Invertebrate diversity	*Chewing damage*	(Jacquot et al. 2019)
Herbivory exclusion	*Rust damage*	(Liu et al. 2021)
Temperature	*Rust damage*	(Chaves et al. 2008)
Litter	*Other fungal damage*	(Zhang et al. 2023)
Invasion	*Other fungal damage*	(Keane and Crawley 2002)

Chaves, Márcia, José Martinelli, C Wesp, and Felipe Graichen. 2008. 'The Cereal Rusts: An Overview'. *Pest Technology* 2 (2): 38–55.

Csurhes, S., C. Leigh, and C. Walton. 2016. 'Weed Risk Assessment: African Lovegrass Eragrostis Curvula'. *Department of Agriculture and Fisheries; Biosecurity Queensland*, no. July: 1–4.

Fricke, Ute, Sarah Redlich, Jie Zhang, et al. 2022. 'Plant Richness, Land Use and Temperature Differently Shape Invertebrate Leaf-Chewing Herbivory on Plant Functional Groups'. *Oecologia* 199 (2): 407–17. https://doi.org/10.1007/s00442-022-05199-4.

Hou, Dongjie, Weiming He, Changcheng Liu, Xianguo Qiao, and Ke Guo. 2019. 'Litter Accumulation Alters the Abiotic Environment and Drives Community Successional Changes in Two Fenced Grasslands in Inner Mongolia'. *Ecology and Evolution* 9 (16): 9214–24. https://doi.org/10.1002/ece3.5469.

Jacquot, Maxime, François Massol, D. Muru, B. Derepas, Philippe Tixier, and Jean Philippe Deguine. 2019. 'Arthropod Diversity Is Governed by Bottom-up and Top-down Forces in a Tropical Agroecosystem'. *Agriculture, Ecosystems and Environment* 285 (March): 106623. https://doi.org/10.1016/j.agee.2019.106623.

Keane, Ryan M., and Michael J. Crawley. 2002. 'Exotic Plant Invasions and the Enemy Release Hypothesis'. *Trends in Ecology and Evolution* 17 (4): 164–70. https://doi.org/10.1016/S0169-5347(02)02499-0.

Klink, R. van, F. van der Plas, C. G.E.Toos van Noordwijk, M. F. Wallisdevries, and H. Olff. 2015. 'Effects of Large Herbivores on Grassland Arthropod Diversity'. *Biological Reviews* 90 (2): 347–66. https://doi.org/10.1111/brv.12113.

Litt, Andrea R., Erin E. Cord, Timothy E. Fulbright, and Greta L. Schuster. 2014. 'Effects of Invasive Plants on Arthropods'. *Conservation Biology* 28 (6): 1532–49. https://doi.org/10.1111/cobi.12350.

Liu, Mu, Tserang Donko Mipam, Xingxing Wang, Peng Zhang, Ziyuan Lin, and Xiang Liu. 2021. 'Contrasting Effects of Mammal Grazing on Foliar Fungal Diseases: Patterns and Potential Mechanisms'. *New Phytologist* 232 (1): 345–55. https://doi.org/10.1111/nph.17324.

Su, Yuan, Kuanhu Dong, Changhui Wang, and Xuejun Liu. 2022. 'Grazing Promoted Plant Litter Decomposition and Nutrient Release: A Meta-Analysis'. *Agriculture, Ecosystems and Environment* 337 (March): 108051. https://doi.org/10.1016/j.agee.2022.108051.

Uetz, W. George. 1979. 'The Influence of Variation in Littler Habitats on Spier Communities'. *Oecologia* 40: 29–42.

Unsicker, Sybille B., Nadine Baer, Ansgar Kahmen, Markus Wagner, Nina Buchmann, and Wolfgang W. Weisser. 2006. 'Invertebrate Herbivory along a Gradient of Plant Species Diversity in Extensively Managed Grasslands'. *Oecologia* 150 (2): 233–46. https://doi.org/10.1007/s00442-006-0511-3.

Vandegehuchte, Martijn L., Wim H. van der Putten, Henk Duyts, Martin Schütz, and Anita C. Risch. 2017. 'Aboveground Mammal and Invertebrate Exclusions Cause Consistent Changes in Soil Food Webs of Two Subalpine Grassland Types, but Mechanisms Are System-Specific'. *Oikos* 126 (2): 212–23. https://doi.org/10.1111/oik.03341.

Zhang, Wei Ping, Dario Fornara, Hao Yang, Rui Peng Yu, Ragan M. Callaway, and Long Li. 2023. 'Plant Litter Strengthens Positive Biodiversity–Ecosystem Functioning Relationships over Time'. *Trends in Ecology and Evolution* 38 (5): 473–84. https://doi.org/10.1016/j.tree.2022.12.008.
